# Centrosomal organization of Cep152 provides flexibility in Plk4 and procentriole positioning

**DOI:** 10.1083/jcb.202301092

**Published:** 2023-09-14

**Authors:** Catherine Sullenberger, Dong Kong, Pegah Avazpour, Delgermaa Luvsanjav, Jadranka Loncarek

**Affiliations:** 1 Cancer Innovation Laboratory, National Institutes of Health, National Cancer Institute, Center for Cancer Research, Frederick, MD, USA

## Abstract

Centriole duplication is a high-fidelity process driven by Polo-like kinase 4 (Plk4) and a few conserved initiators. Dissecting how Plk4 and its receptors organize within centrosomes is critical to understand the centriole duplication process and biochemical and architectural differences between centrosomes of different species. Here, at nanoscale resolution, we dissect centrosomal localization of Plk4 in G1 and S phase in its catalytically active and inhibited state during centriole duplication and amplification. We build a precise distribution map of Plk4 and its receptor Cep152, as well as Cep44, Cep192, and Cep152-anchoring factors Cep57 and Cep63. We find that Cep57, Cep63, Cep44, and Cep192 localize in ninefold symmetry. However, during centriole maturation, Cep152, which we suggest is the major Plk4 receptor, develops a more complex pattern. We propose that the molecular arrangement of Cep152 creates flexibility for Plk4 and procentriole placement during centriole initiation. As a result, procentrioles form at variable positions in relation to the mother centriole microtubule triplets.

## Teaser

Sullenberger et al. dissect the centrosomal patterning of Plk4 during centriole duplication and amplification and the positioning of Plk4 and procentrioles in relation to mother centriole microtubules. They build a molecular map of Cep57, Cep63, Cep192, and Cep152 and investigate their requirements for Plk4 localization.

## Introduction

In human cycling cells, centrosomes nucleate microtubules, organize mitotic spindle poles, form cilia, and mediate signaling, amongst other functions (Arquint et al., 2014; Bornens, 2021; Nigg and Holland, 2018). The core of a centrosome is a centriole, a ninefold symmetrical microtubule-based cylindrical structure (LeGuennec et al., 2021; Vorobjev and Chentsov, 1982). Centrioles duplicate only once during the cell cycle, upon S phase entry, when just one new procentriole forms in the vicinity of each of the two resident mother centrioles (Nigg and Holland, 2018). Deviations in the centriole duplication cycle such as assembly of multiple procentrioles (i.e., amplification) or multiple rounds of duplication during interphase (i.e., reduplication) result in aberrant centrosome number with negative consequences on cell and tissue homeostasis (Arnandis et al., 2018; Basto et al., 2008; Levine et al., 2017; LoMastro and Holland, 2019; Nigg et al., 2017).

Polo-like kinase 4 (Plk4; Habedanck et al., 2005), SAK in *Drosophila melanogaster* (Bettencourt-Dias et al., 2005), and Zyg-1 in *Caenorhabditis elegans* (O’Connell et al., 2001) is a central regulator of centriole initiation. On unduplicated mother centrioles in G1 phase, using resolution-limited imaging or structured illumination microscopy, Plk4 appears as a ring around the proximal end of mother centrioles. At the onset of centriole duplication, Plk4 changes to an asymmetric signal (Kim et al., 2013; Ohta et al., 2014), where it colocalizes with other centriole initiating factors STIL (Tang et al., 2011; Vulprecht et al., 2012; Ana2 in *Drosophila* [Dobbelaere et al., 2008; Goshima et al., 2007; Stevens et al., 2010]; SAS-5 in *C. elegans* [Delattre et al., 2004]), and SAS-6 (Strnad et al., 2007), marking the site of procentriole initiation. Although Plk4 robustly localizes to the centrioles in G1, centriole duplication does not occur before STIL (Arquint and Nigg, 2014; Arquint et al., 2012; Tang et al., 2011) and SAS-6 (Strnad et al., 2007) accumulate in S phase, allowing Plk4 and STIL interaction, which promotes Plk4 kinase activity (Moyer et al., 2015). Human Plk4 is reportedly localized to centrosomes through interaction with two receptors, Cep192 and Cep152 (Cizmecioglu et al., 2010; Dzhindzhev et al., 2010; Hatch et al., 2010; Kim et al., 2013; Sonnen et al., 2013). While Cep192 is present on centrioles in their first cell cycle (Fig. 3 C; Tsuchiya et al., 2016), Cep152 associates with nascent centrioles after disengagement in their first G1 (Kim et al., 2013; Park et al., 2014). Associated proteins Cep57 and Cep63, which bind to parental centriole microtubules and Cep152, respectively, indirectly assist in Plk4 loading to the centrosome (Brown et al., 2013; Kim et al., 2019; Lukinavicius et al., 2013; Sir et al., 2011; Wei et al., 2020). Molecular assemblies of Cep57 and Cep63–Cep152 complexes generate a scaffold that is critical for centriole initiation and centriole duplication control (Lee et al., 2020; Wei et al., 2020). While binding properties of Plk4 and its receptor molecules have been extensively dissected biochemically, their centrosomal organization, as well as the organization of Cep57 and Cep63, still requires dissection at high resolution.

In this work, we combine sample expansion, STED imaging, and electron microscopy to dissect centrosomal patterning of Plk4 during centriole duplication and amplification and Plk4 inactivation in G1 and S phase cells. We further analyze Plk4 and procentriole positioning with respect to the mother centriole circumference. In addition, we unravel a molecular map of Cep57, Cep63, Cep192, and Cep152, and investigate their requirements for Plk4 localization.

## Results and discussion

**Figure S1.**
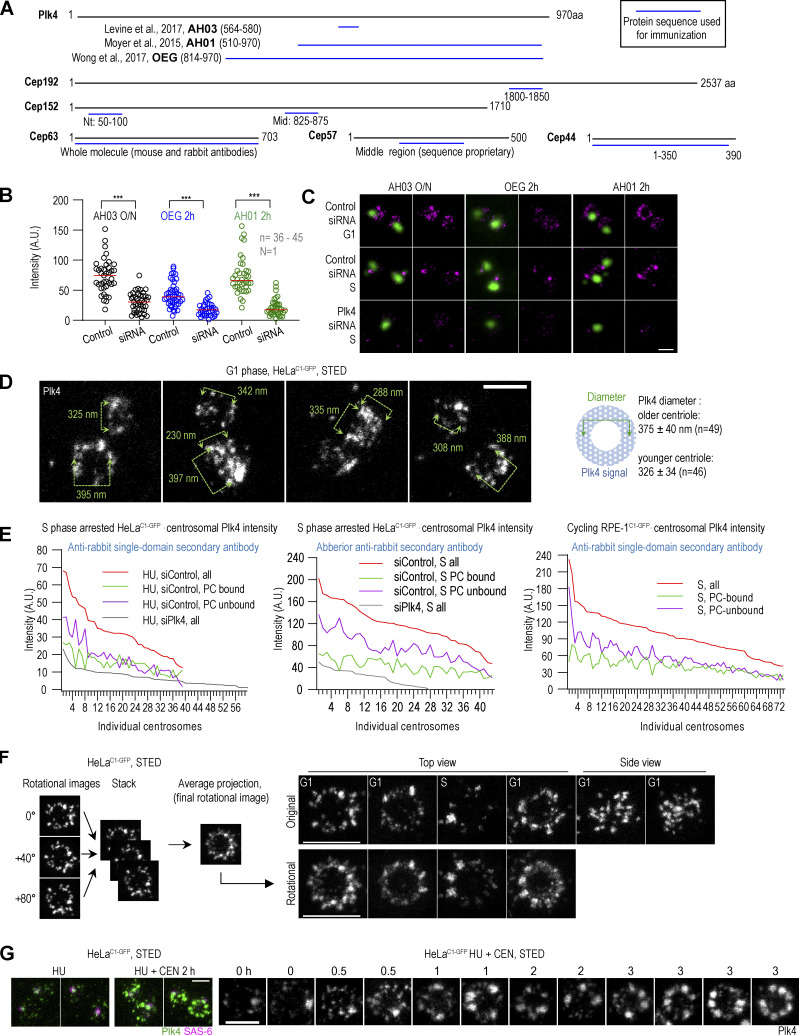
**Antibodies against Plk4 and other proteins and centrosomal Plk4 organization. (A)** Schematic of peptides (blue lines) used to generate antibodies used in this paper. Sources and antibody nomenclature (bold letters) for custom Plk4 antibodies are indicated. **(B and C)** Cycling HeLa^C1-GFP^ cells were synchronized by mitotic shake-off and treated with Plk4 siRNA for 40 h. Plk4 was immunolabeled with indicated Plk4 antibodies for 2 h at 37°C or overnight at 4°C (O/N), followed by incubation with fluorescently labeled secondary antibodies. **(B)** Quantification of centrosomal Plk4 signals from STED images. In Plk4 siRNA-treated samples, only cells with one centrosome were included in the analysis to ensure that Plk4 was depleted. Box and whisker plot shows the minimum and maximum value, median, upper, and lower quartiles, and all data points, including outliers. *n* = number of centrosomes; *N* = number of independent experiments. **(C)** STED images of Plk4 immunolabeled centrosomes in non-depleted and depleted cells. Centrin1-GFP signal marks centrioles’ position. **(D)** Plk4 was immunolabeled in a population of G1 cells, and centrosomal Plk4 was imaged using STED. The numbers on the figure panels indicate the dimensions of the Plk4 signal between the points indicated by arrows. The centriole containing more Plk4 is likely the older centriole. The average diameter ± SD of Plk4 signals of older and younger centrioles is indicated. **(E)** S phase arrested HeLa^C1-GFP^ and cycling RPE-1^C1-GFP^ cells were fixed and immunolabeled for Plk4 and imaged by STED. The total, procentriole (PC)-bound, and PC-unbound Plk4 was quantified, and the data was plotted for individual centrosomes. **(F)** The left panel illustrates the strategy used to generate rotational images of centrosomal signals of centrioles imaged in vertical or near-vertical orientation with respect to the imaging plane. Centrioles were centered in the middle of the canvas. Three rotational images of the centrioles were generated by rotating images for 0°, 40°, and 80°, assembled in a stack, and an average projection was made. Right: Original and rotated images of Plk4 signals from cycling cells. **(G)** S phase arrested HeLa^C1-GFP^ cells were treated with Plk4 inhibitor CEN for up to 3 h, immunolabeled for Plk4 and SAS-6 (procentriole marker), and centrosomes were analyzed by STED. HU = hydroxyurea. Scale bars: 0.5 µm for STED and 2 µm for fourfold expansion + STED.

To dissect the localization pattern of Plk4 and other centrosomal proteins in G1 and S phases in HeLa, U2OS, and RPE-1 cells, we employed immunolabeling, cell expansion, and STED microscopy. We immunolabeled samples prior to expansion and used secondary antibodies conjugated with a STED dye, which is highly tolerant to the expansion procedure (Vásquez-Limeta et al., 2022). We first focused on the levels and localization of Plk4 related to its previously reported ring-to-dot transformation during the G1-S transition. We first compared immunolabeling signals obtained by three published, custom Plk4 antibodies (Fig. S1 A). All antibodies were sensitive to the loss of Plk4 after siRNA treatment and showed similar immunolabeling patterns at G1- and S-phase centrosomes (Fig. S1, B and C). The AH01 antibody (Moyer et al., 2015) showed the greatest specificity toward Plk4 and was used for most of this work, though comparable results were produced with other antibodies.

**Figure 1.**
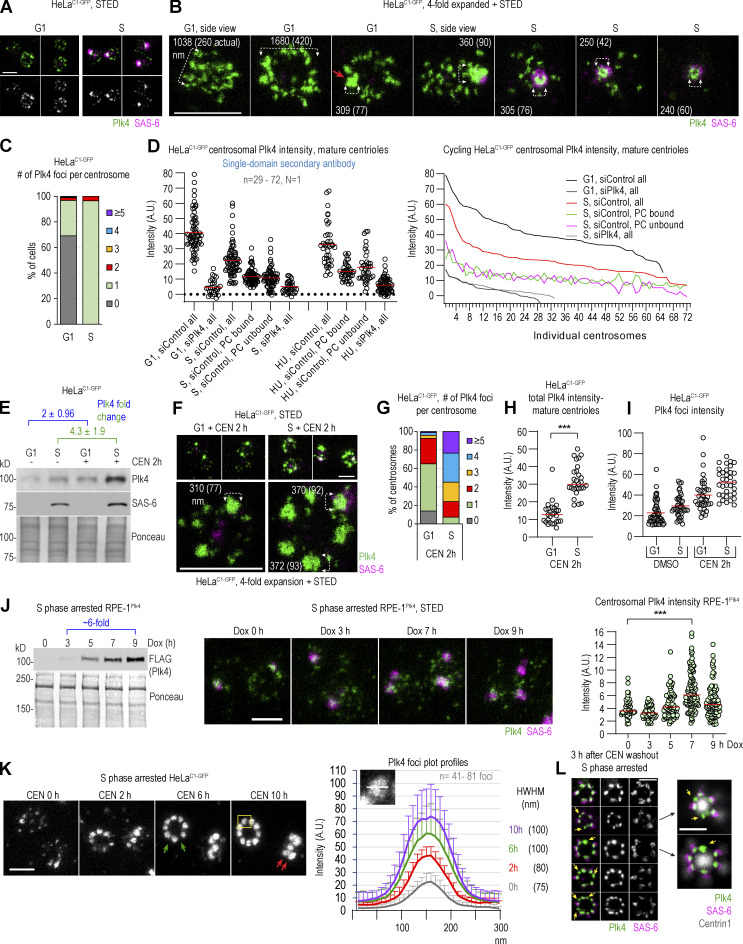
**Centrosomal distribution of Plk4 in its active and catalytically inhibited state. (A–I)** Cycling G1 or S phase HeLa^C1-GFP^ cells were treated with the Plk4 inhibitor CEN or DMSO (control) for the indicated time and immunolabeled for Plk4 and procentriole cartwheel protein SAS-6. **(A and B)** Distribution of centrosomal Plk4 and SAS-6 from control cells. **(C)** Quantification of Plk4 foci on centrosomes in G1 and S. **(D)** Quantification of the total, procentriole (PC)-bound, and PC-unbound Plk4 quantified from STED images. Line plot: The same data plotted as individual centrosomes. **(E)** Immunoblot analysis of Plk4 and SAS-6 from total cell extracts. Ponceau serves as a loading control. **(F)** Distribution of centrosomal Plk4 and SAS-6 in CEN-treated cells. **(G)** Quantification of Plk4 foci number per centrosome in CEN-treated cells. **(H)** Total Plk4 intensity on mature centrosomes quantified from STED images. **(I)** The intensity of individual Plk4 foci quantified from STED images. **(J)** Immunoblot of FLAG-Plk4 signal from total cell lysates. Expression of FLAG-Plk4 was induced by Dox for 0–9 h. Ponceau serves as a loading control. Middle: Images of centrosomes obtained from parallel samples. Right: Total Plk4 intensity on centrosomes quantified from STED images. The red line: average intensity. **(K)** STED images of immunolabeled Plk4 at centrosomes were recorded and used to measure intensity profiles for individual Plk4 foci (as shown in insert). Averaged intensity ± SD for each time point is plotted. The average size of Plk4 foci was determined at half-width–half-maxima of the plot profile. The green arrows indicate variable distances between adjacent Plk4 foci around the mother centriole. The red arrows point to Plk4 foci at different lateral positions along the mother centriole. **(L)** Plk4 foci and their association with SAS-6 after CEN washout. The yellow arrows point to Plk4 foci not associated with SAS-6. *N* = the number of independent replicates presented. *n* = the number of quantified centrosomes or Plk4 foci. Scale bars: 0.5 µm for STED and 2 µm for fourfold expansion + STED. Source data are available for this figure: SourceData F1.

As previously reported (Kim et al., 2013; Ohta et al., 2014; Park et al., 2014), in G1, centrosomal Plk4 occupies a cylinder around centrioles (Fig. 1, A and B). In mature centrioles, the cylinder was ∼260 nm long and ∼375 nm wide (measuring through its central diameter; Fig. S1 D). One G1 centriole, presumably the younger one, harbored less Plk4 (Fig. 1 A, Fig. S1 D, and Park et al., 2014). In ∼25% of G1 centrosomes, one or two brighter and larger Plk4 foci could be detected within the Plk4 occupying volume (Fig. 1 B, red arrow, and Fig. 1 C). Since singular Plk4 foci were detected only on a minority of G1 centrosomes (including late G1 centrosomes), we reasoned that these foci, although they might serve as the sites for future centriole initiation in early S, are not critical for centriole copy control or centriole initiation. In S phase, a distinguishable, discrete Plk4 signal colocalized with SAS-6, as typical for centrosomes with duplicated centrioles (Kim et al., 2013; Ohta et al., 2014), with additional smaller Plk4 signals of lower intensity variably detected around mother centrioles (Fig. 1, A and B). SAS-6 associated Plk4 foci were usually larger and brighter than Plk4 foci detected on G1 centrosomes (Fig. 1, A, B, and I [compare DMSO G1 centrosomes to SAS-6-positive S phase centrosomes]), although there were exceptions.

We did not detect a continuous ring of Plk4 in any control G1 or S phase cells. Rather, numerous Plk4 signals could be found in isolation, surrounded by signal-less areas. Plk4 signal did not seem to follow the ninefold symmetrical pattern of mother centrioles either. We further explored this by averaging three images of the same centrosome rotated for 0°, 40°, and 80° (Fig. S1 F), but rotational averaging failed to reveal a clear symmetrical pattern of Plk4. In addition, in longitudinally oriented centrioles, the Plk4 signal appeared unorderly and without a linear pattern, which would be expected at our achieved resolution if ninefold symmetry was present, as we later document for ninefold symmetrically organized proteins (Fig. S3, A and C).

On average, mature centrioles of G1 HeLa^C1-GFP^ cells harbored more total Plk4 than S phase centrosomes (Fig. 1 D; despite lower cytosolic levels of Plk4 in G1 cells; Fig. 1 E), indicating that at the G1/S transition, a portion of Plk4 is lost and not simply reorganized within the Plk4-occupying cylinder. To understand which amount of total centrosomal Plk4 on S phase centrosomes associates with procentrioles, we specifically measured Plk4 associated with the SAS-6 cartwheel (Fig. 1 D). In Hela^C1-GFP^ S phase cells, on average, ∼50% of total Plk4 was procentriole bound, irrespective of whether Hela^C1-GFP^ cells were cycling or arrested in S phase. A similar ratio was detected on centrosomes in cycling RPE-1 cells (Fig. S1 E, right panel). Plotting Plk4 values of individual centrosomes (Fig. 1 D, line plot, and Fig. S1 E) revealed a positive correlation between total and procentriole-bound Plk4 levels. It also made it obvious that the levels of procentriole-unbound Plk4 differ significantly between centrosomes, even several-fold amongst measured centrosomes. At some S phase centrosomes, procentriole-unbound Plk4 even exceeded the total Plk4 levels of mature G1 centrosomes (Fig. 1 D). Procentriole-unbound Plk4 was more pronounced if Plk4 was detected using conventional secondary antibodies than with single-domain antibodies (compare the left and the middle line plots in Fig. S1 E). Plk4 quantification results aligned with our microscopy analysis (Fig. 1 B, S phase centrosomes), in which we routinely identified centrosomes with variably abundant procentriole-unbound Plk4 signal. However, we did not observe any instances where this procentriole-unbound Plk4 induced the formation of multiple procentrioles around mother centrioles. Consequently, we infer that the centriole-intrinsic block to reduplication (Tsou and Stearns, 2006; Wong and Stearns, 2003) can be efficiently maintained on all centrosomes and that it can operate in the presence of substantial levels of non-procentriolar Plk4. Perhaps, a tight control of centriole copy number involves other parameters, in addition to controlled stoichiometry of centrosomal Plk4. Or the non-procentriolar Plk4 fraction could be inactive or represent posttranslationaly modified Plk4 species destined for degradation or dissociation and unable to bind STIL.

Inhibition of catalytic Plk4 activity by centrinone (CEN; Wong et al., 2015) increased cytosolic and centrosomal levels of Plk4 (Fig. 1, E and F; and Wong et al., 2015) and promoted the formation of additional Plk4 foci of increased brightness around mother centrioles (Fig. 1, F–I; and Takao et al., 2019). Although inactive Plk4 foci formed in both G1 and S phase cells, their number was significantly higher in S phase (Fig. 1 G). We reasoned that this could be due to the higher accumulation of cytosolic Plk4 in CEN-treated S phase cells (Fig. 1 E) or differences in the regulation of centrosomal Plk4 loading or turnover between G1 and S phases. Inhibition of Plk4 in S phase-arrested cells resulted in a similar phenotype (Fig. S1 G). Catalytically inactive Plk4 foci formed gradually after CEN addition (Fig. S1 G). After ∼30 min, the number of smaller, intermittently distributed signals increased around centrioles. With time, these signals increased in size and intensity, and after longer treatment, a variable number (1–10) of Plk4 emerged around mother centrioles. Low-intensity, unstructured signals were still present between Plk4 foci.

**Figure S2.**
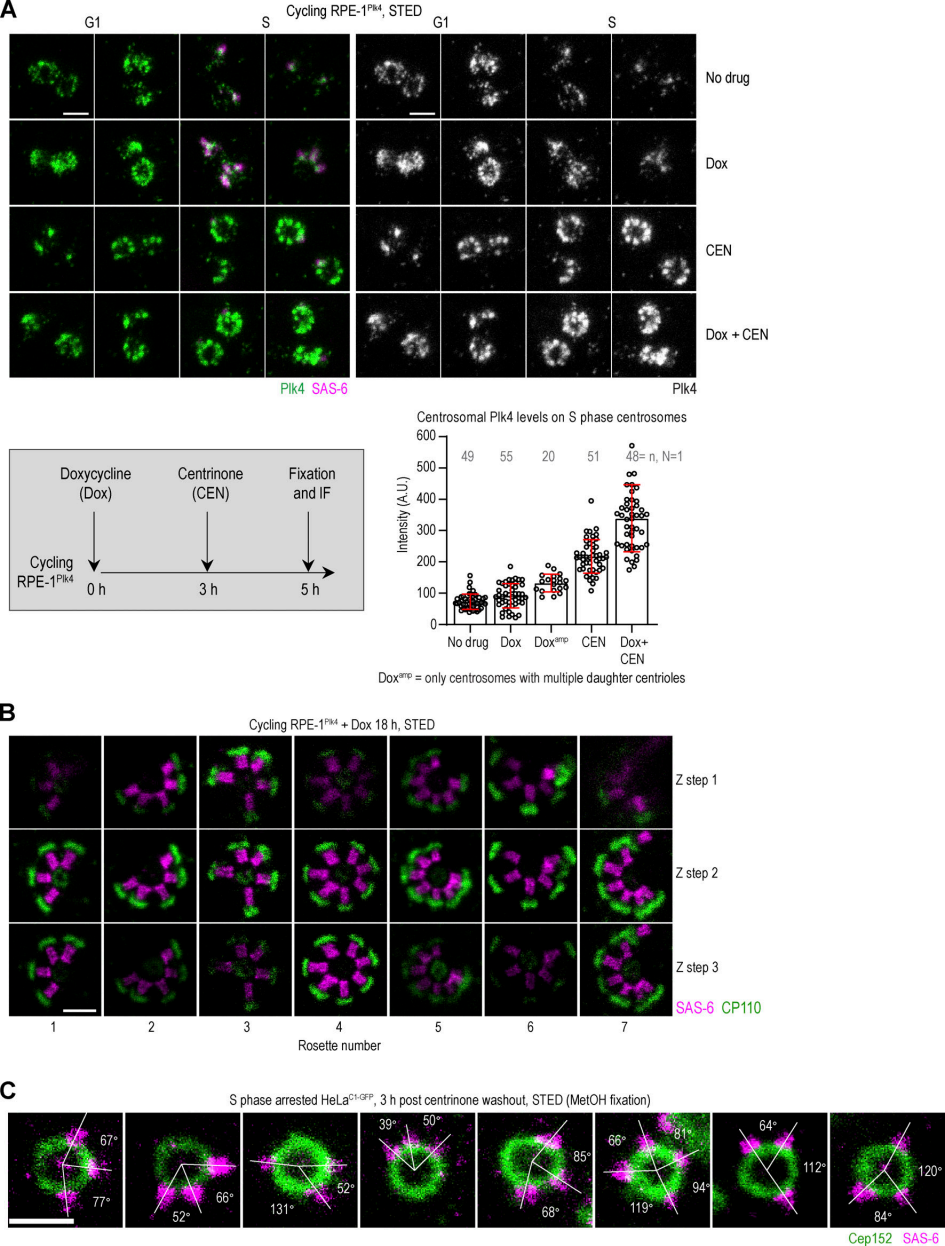
**Centrosomal organization of catalytically uninhibited and inhibited overexpressed Plk4 and centriolar rosettes. (A)** Centrosomal organization of overexpressed Plk4 in the absence and in the presence of its catalytic inhibitor CEN. S phase arrested RPE-1^Plk4^ cells were treated as depicted in the gray box. Dox was added to induce Plk4 expression, after which CEN was added to inhibit Plk4. Cells were fixed and immunolabeled for Plk4 and SAS-6 and imaged using STED. The centrosomal intensity of Plk4 was measured from STED images and plotted. The plot shows all individual points and average ± SD. *n* = number of centrosomes; *N* = number of independent experiments. **(B)** Three Z sections of centriolar rosettes from Plk4-overexpressing RPE-1^Plk4^ cells. The middle Z section is shown in Fig. 2 C. CP110 labels centriole distal ends and SAS-6 labels procentriole cartwheels. **(C)** Organization of centriolar rosettes induced by a transient CEN treatment after fixation in methanol (MetOH). S phase arrested cells were treated with CEN for 3 h, after which CEN was washed out to allow the formation of procentrioles around mother centrioles for 3 h. Cells were immunolabeled for Cep152 and SAS-6 and imaged by STED. White lines connect the centers of SAS-6 foci and the physical center of the mother centriole. The angles measured between two adjacent lines are indicated. Scale bars: 0.5 µm.

When we induced overexpression of Plk4 in cycling RPE-1^Plk4^ cells by doxycycline (DOX), without inhibition of its catalytic activity, cytosolic levels of Plk4 increased and centrioles amplified (Fig. 1 J and Habedanck et al., 2005; Kleylein-Sohn et al., 2007). However, even with significantly increased cytosolic Plk4 levels, robust foci failed to form on centrosomes (Fig. 1 J), contrary to what we observed in CEN-treated cells. Consistently, total centrosomal Plk4 levels only moderately increased, even on centrosomes with amplified centrioles (Fig. 1 J and Fig. S2 A). On centrosomes with amplified centrioles, the modest increase of centrosomal Plk4 was likely due to Plk4 bound to multiple procentrioles. Catalytic inhibition of overexpressed Plk4 by CEN resulted in further centrosomal accumulation of Plk4 and the formation of robust Plk4 foci, both in G1 and S phase cells (Fig. S2 A). This data signifies a faster centrosomal turnover of active Plk4 compared with catalytically inhibited Plk4, consistent with other work (Yamamoto and Kitagawa, 2019). It also suggests that centriole amplification occurs without extensive Plk4 focusing seen after CEN treatment. In agreement, we observed that de novo centrioles formed after Plk4 overexpression usually contain minimal levels of Plk4 associated with their proximal ends (data not shown). Therefore, a more precise definition of what constitutes a Plk4 focus/aggregate is warranted.

The foci of inhibited Plk4 appeared similar in HeLa cells and RPE-1 cells with overexpressed Plk4 (Fig. 1 F, Fig. S1 G, and Fig. S2 A). To further explore Plk4 focusing, we analyzed them after a longer (10 h) period of Plk4 inhibition in HeLa^C1-GFP^ cells synchronized in S phase. Within the first 6 h of inhibition, foci reached the size of ∼100 nm, after which they maintained a similar size. Their average intensity increased ∼twofold within the first 2 h and continued to increase but at a slower rate until 10 h. Foci also formed at different lateral positions of the Plk4-occupying cylinder (Fig. 1 K, red arrows). In the top view, a variable number of Plk4 foci (1–10) were arranged in a circular fashion, but their mutual distance appeared irregular on many centrosomes (Fig. 1 K, green arrows). After CEN washout, ∼90% of Plk4 foci had initiated centrioles, judging by the appearance of SAS-6 and nascent C1-GFP signals around mother centrioles (Fig. 1 L). Foci that failed to associate with SAS-6 persisted around the centrioles for at least 3 h (Fig. 1 L, yellow arrows). Longer time points were not explored.

The uneven distribution of Plk4 foci and the notion that Plk4 foci could form at different lateral positions along the proximal end of mother centrioles prompted us to analyze their position in relation to mother centriole microtubules.

**Figure 2.**
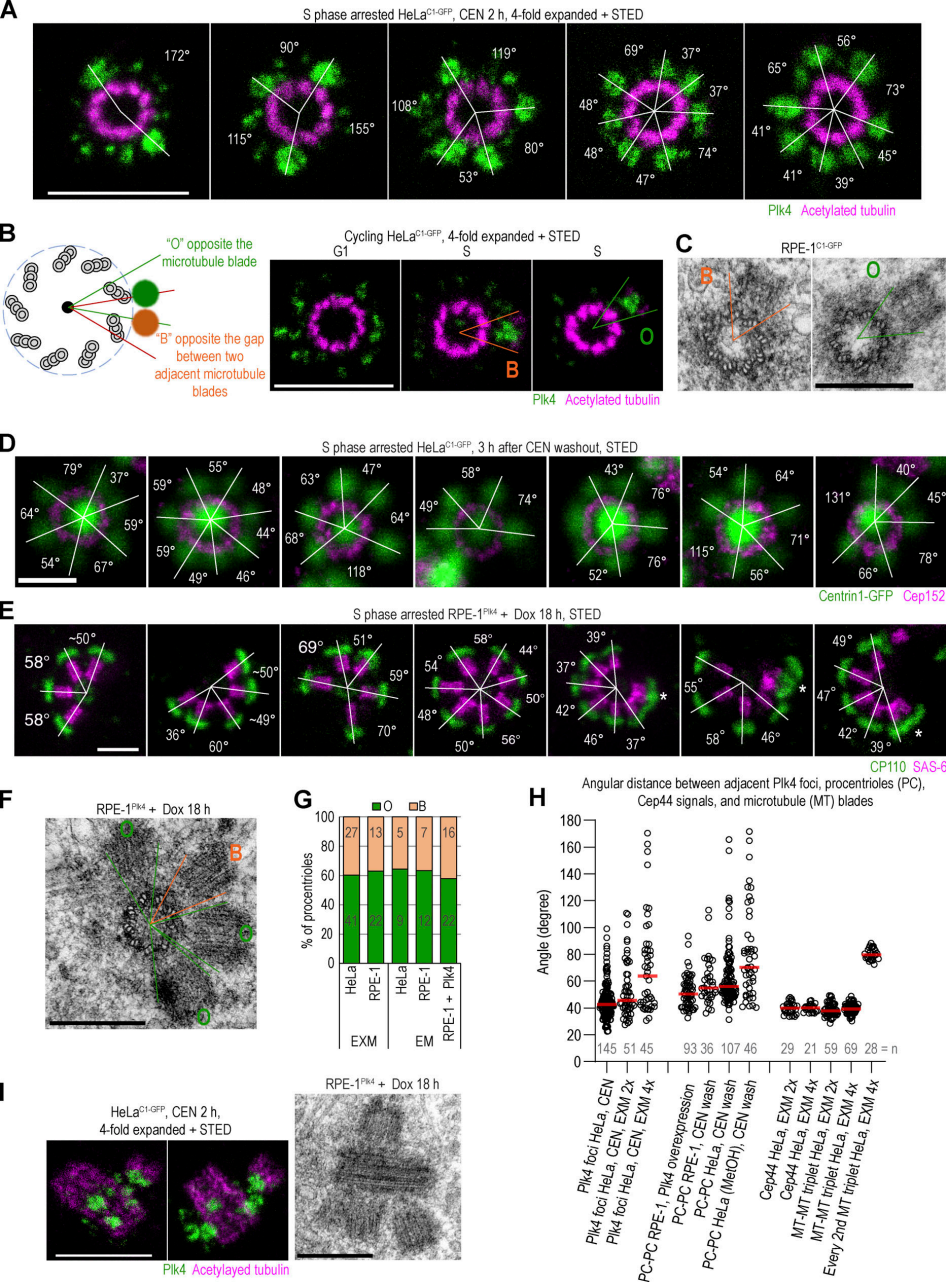
**Localization of Plk4 foci and procentrioles in relation to the mother centriole microtubule wall. (A)** Images of Plk4 foci around centrioles after 2 h of CEN treatment. Samples were immunolabeled for Plk4, expanded, and postlabeled for acetylated tubulin. White lines connect the centers of Plk4 foci and the physical center of the mother centriole. The angles measured between two adjacent lines are indicated. **(B)** A schematic depicting two positions Plk4 foci and procentrioles occupy with respect to mother centriole microtubules. O = facing opposite microtubule blades. B = facing opposite the gap between microtubule triplets. Right: Examples of Plk4 foci/procentrioles in the B and O positions. **(C)** Electron micrographs of duplicated centrioles from RPE-1^C1-GFP^ cells. One procentriole is in B and another in the O position. **(D)** Centriolar rosettes after transient CEN treatment. Cells were treated for 3 h, after which CEN was washed out to allow the formation of procentrioles around mother centrioles. Centrin1-GFP marks centrioles. Cep152 labeling was used to confirm the orthogonal orientation of mother centrioles. White lines and angles as in A. **(E)** Centriolar rosettes from Dox-induced Plk4-overexpressing RPE-1^Plk4^ cells. CP110 labels centriole distal ends and SAS-6 procentriole cartwheels. White lines and angles, as in A. Asterix marks overlapping procentrioles formed at different lateral highs. **(F)** Electron micrograph of a centriolar rosette from Plk4-overexpressing RPE-1^Plk4^ cells. **(G)** Quantification of procentrioles’ position around mother centriole. **(H)** Quantification of angles between adjacent Plk4 foci and procentrioles from various experiments. The angles were determined as indicated in A. Cep44 and acetylated tubulin signals exhibit an average angular distance of 40°, consistent with their symmetrical ninefold pattern, and were used to validate the quantification approach. **(I)** Plk4 and procentrioles at different lateral positions along the mother centriole longitudinal axis obtained by CEN treatment (STED images) or Dox-induced Plk4 overexpression (electron micrograph). Scale bars: 0.5 µm for STED, 2 µm for fourfold expansion + STED, and 0.4 µm for electron micrographs.

We colabeled Plk4 and acetylated tubulin to mark centriole microtubules (Sahabandu et al., 2019; Vásquez-Limeta et al., 2022) and imaged centrioles oriented vertically or near-vertically to the plane of imaging. In CEN-treated HeLa cells, measuring from the center of the focus to the nearest acetylated tubulin signal of the mother centriole, we observed that Plk4 foci largely faced either a single MT blade or the gap between two adjacent MT blades of the mother centriole wall (Fig. 2, A and B). Determining radial angles through the centers of adjacent foci and the mother centriole center revealed that many adjacent foci form at an angle of ∼40° and increments of 40°, consistent with ninefold symmetry. However, we also regularly measured intermediate angles (Fig. 2, A and H), suggesting that the entire mother centriole perimeter is permissive for Plk4 loading and focusing. We questioned whether this flexibility in Plk4 focusing translates to flexibility, in which procentrioles assemble with respect to mother centriole microtubules. Indeed, both expansion/STED and electron microscopy revealed that procentrioles’ proximal ends can face a single microtubule blade or the gap between two adjacent blades of the mother centriole wall (Fig. 2, B and C). Moreover, procentrioles in centriolar rosettes formed after CEN-washout or Plk4 overexpression also formed at variable radial angles around the mother centriole circumference (Fig. 2, D, E, and H; and Fig. S2, B and C) and can face either the microtubule blade or the gap between two adjacent blades of the mother centriole (Fig. 2 F). Nearly 60% of imaged procentrioles faced the microtubule blade and ∼40% faced the gap between the two blades (Fig. 2 G). Notably, procentriolar rosettes formed around mother centrioles of mouse tracheal epithelial multiciliated cells follow the same pattern (Nanjundappa et al., 2019). Thus, there is flexibility in the positioning of Plk4 and, hence, procentrioles relative to the mother centriole MT blades on vertebrate centrosomes. In addition, we document that Plk4 focusing and procentriole formation after Plk4 overexpression can occur at variable lateral positions around mother centrioles (Fig. 2 I).

**Figure 3.**
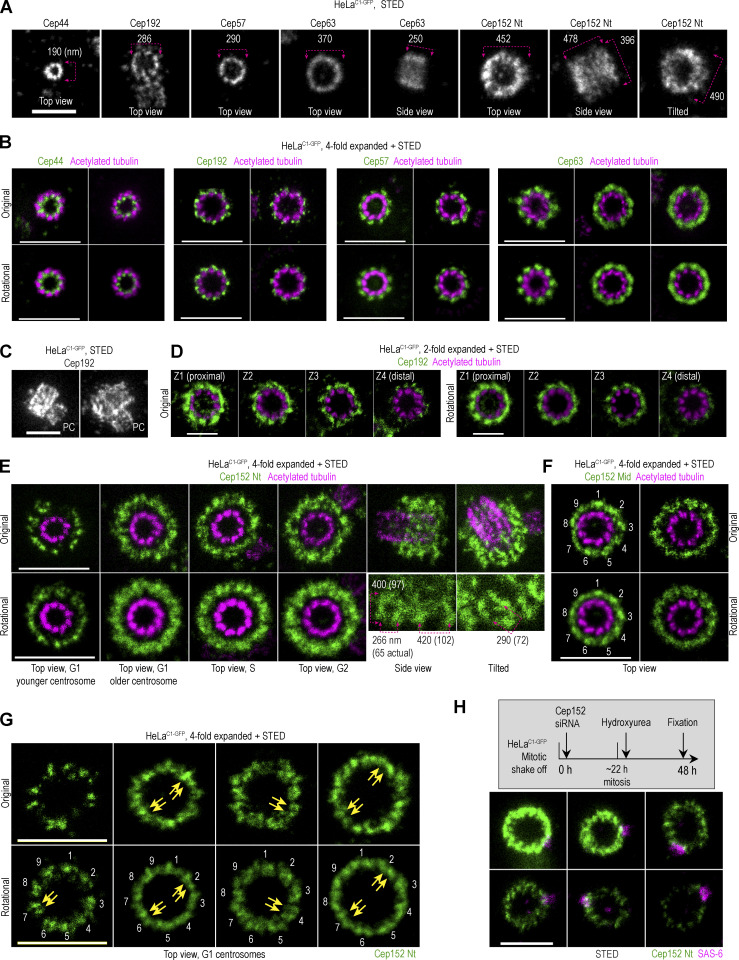
**Spatial organization of proximal centriole proteins in HeLa cells.** Logarithmically growing HeLa^C1-GFP^ cells were immunolabeled with antibodies to detect Cep44, Cep192, Cep57, Cep63, or Cep152. Some samples were additionally expanded and labeled for acetylated tubulin. Imaging was done using STED. Rotational images of centrioles in the top view were additionally generated by averaging three images of the same centrosome rotated for 0°, 40°, and 80° (as depicted in Fig. S1 F). **(A)** Examples of fluorescent signals for various proteins. Nt = N-terminus. The outer diameter or lateral dimensions of the signals are indicated. **(B)** Original and averaged images of expanded centrioles imaged in the top view. **(C)** Images of centrosomes with duplicated mother centrioles immunolabeled for Cep192, which is localized on mother centrioles and procentrioles (PC). **(D)** Four Z sections of vertically oriented centrosomes illustrate different levels of Cep192 along the mother centriole. **(E and F)** Examples of expanded centrosomes with immunolabeled Cep152 using antibodies against the Nt or middle region (Mid; Fig. S1 A) and acetylated tubulin. Numbers in F indicate denser portions of the signal. **(G)** G1 centrosomes immunolabeled for Cep152 Nt. Numbers indicate nine groups of signals, with two adjacent signals within (some indicated by arrows). **(H)** Organization of Cep152 on centrosomes with various degrees of Cep152 depletion. Cep152 was depleted in HeLa^C1-GFP^ cells, as depicted in the gray box. Cells were immunolabeled for Cep152 and procentriole marker SAS-6. Scale bars: 0.5 µm for STED, 1 µm for twofold expansion + STED, and 2 µm for fourfold expansion + STED.

To understand how stringent ninefold centriolar symmetry results in flexible procentriole positioning, we next investigated the localization pattern of Plk4’s reported centrosomal receptors Cep192 and Cep152 (Cizmecioglu et al., 2010; Dzhindzhev et al., 2010; Hatch et al., 2010; Kim et al., 2013; Sonnen et al., 2013), and Cep152 anchoring proteins Cep57 and Cep63 (Brown et al., 2013; Kim et al., 2019; Lukinavicius et al., 2013; Sir et al., 2011; Wei et al., 2020). In parallel, we analyzed Cep44, a centriole luminal protein that is needed for centriole structuring and maturation, and, in turn, for Cep192 and Cep152 localization (Atorino et al., 2020). We visualized proteins using validated commercial antibodies, as described in Fig. S1 A. In HeLa cells (Fig. 3 A), all proteins formed a cylinder with a ring-like organization when viewed from the top, as previously reported (Kim et al., 2013; Lawo et al., 2012; Lukinavicius et al., 2013; Mennella et al., 2012; Park et al., 2014; Sonnen et al., 2013; Sonnen et al., 2012; Wei et al., 2020). We determined the dimensions of these cylinders from non-expanded samples by STED and later used these values to confirm the consistency of sample expansion. For expanded centrioles, imaged in a vertical or near-vertical orientation, we present the original recordings and their averaged signals obtained from rotating and averaging each image for 0°, 40°, and 80°, as described earlier for Plk4 (Fig. S1 F).

**Figure S3.**
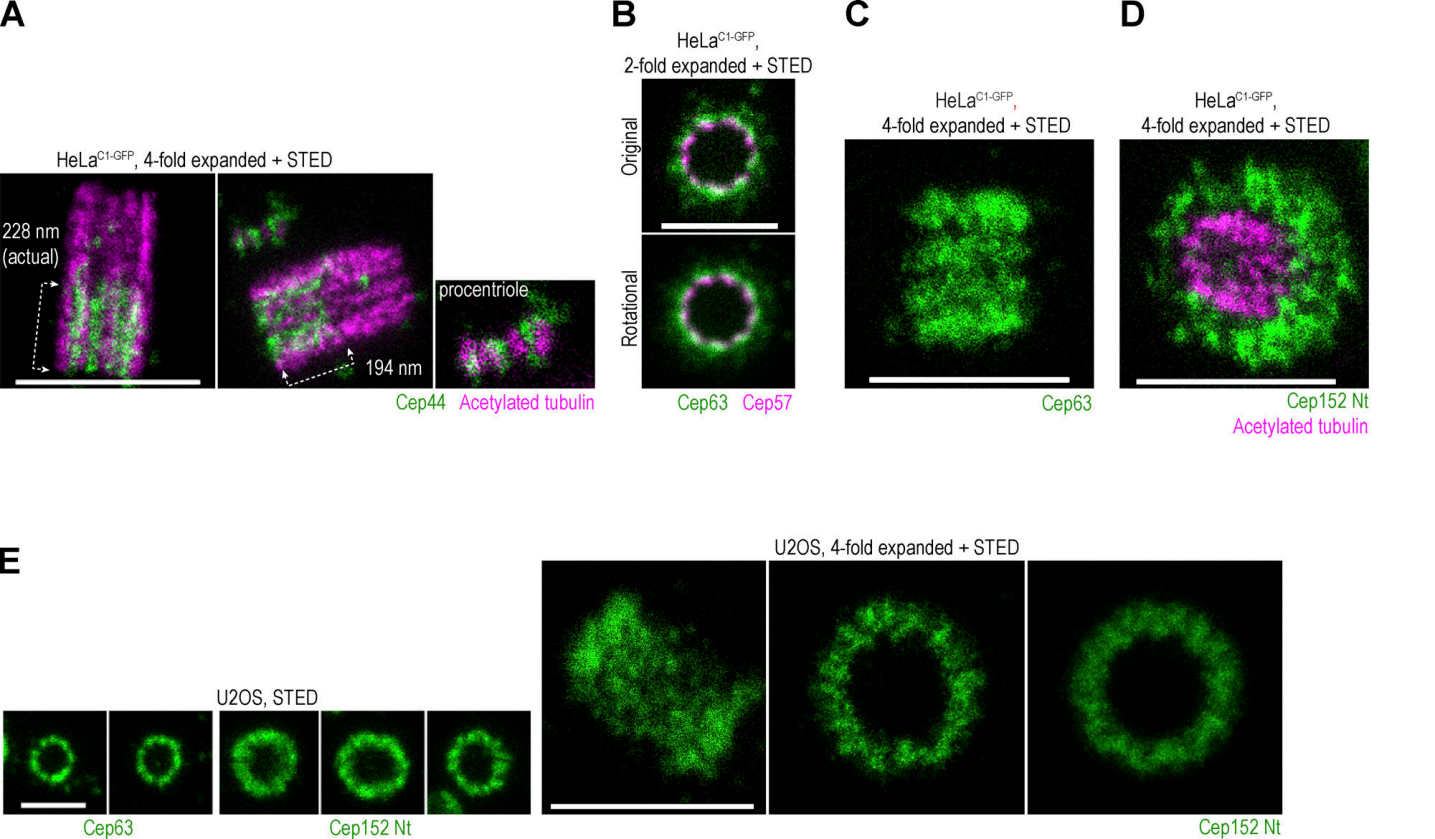
**Spatial arrangement of various centrosomal components. (A–E)** Cells were immunolabeled with indicated antibodies and expanded. Some samples were additionally labeled for acetylated tubulin. Centrosomes were imaged using STED. **(A)** Longitudinally oriented centrosomes immunolabeled for Cep44 and acetylated tubulin. Cep44 localizes to procentrioles from their early stages of formation. **(B)** STED images of centrioles in top view, co-labeled with Cep63 and Cep57. The two proteins are in proximity, consistent with biochemical analyses showing that they interact. **(C)** Image of longitudinally imaged centriole immunolabeled for Cep63 signals, showing the distribution of Cep63 in a linear fashion, consistent with its ninefold radial distribution around mother centrioles. **(D)** Image of a centriole tilted toward the imaging plane and immunolabeled for Cep152 and acetylated tubulin to illustrate the three-dimensional arrangement of the Cep152 signal. **(E)** Images of Cep63 and Cep152 from U2OS cells show a similar arrangement as found in HeLa cells. Scale bars: 0.5 µm for STED, 1 µm for twofold expansion + STED, and 2 µm for fourfold expansion + STED.

Cep44 formed nine discrete signals organized in a ring of ∼190 nm (Fig. 3, A and B). Expansion and colabeling with acetylated tubulin revealed that the Cep44 signal localizes at the base of microtubule triplets (adjacent to the inner, A, microtubule), and in some centrioles, extends within the space between two microtubule blades. Cep44 was present in centrioles from their early stages, and on mature centrioles, the length of the Cep44 cylinder was 201 ± 78 nm (*n* = 27; Fig. S3 A).

In the top view, nine Cep192 signals were organized in a ring of ∼286 nm in diameter (Fig. 3, A and B). Cep192 levels varied among centrosomes (Fig. 3, C and D). Consequently, the gap between the lobules was less evident on some centrosomes. When ninefold symmetry was evident, most of the Cep192 localized outside the centriolar cylinder, adjacent to the outer C tubule of a microtubule triplet (Fig. 3 B). It followed the chirality of centriole microtubule blades and, looking from the centriole’s distal end, followed the right turn of the centriole microtubules. Unlike other herein-analyzed proteins which occupy a cylindrical space toward the proximal end of centrioles, Cep192 localizes along the entire centriole length (Fig. 3 C; Sonnen et al., 2013). Z sectioning through expanded centrioles showed that Cep192 preferentially localizes adjacent to the outer microtubule along the entire centriole length (Fig. 3 D). When more abundant, Cep192 also occupied the space opposite to microtubule blades.

Cep57 was organized in nine distinguishable lobules in a ring of ∼290 nm in diameter (Fig. 3 A). Its lobules were positioned adjacent and opposite to the middle of the microtubule blades. The levels of Cep57 did not differ significantly between centrosomes. Cep57 binding partner Cep63 formed a ring of ∼370 nm in diameter and ∼250 nm in length (Fig. 3A), which consisted of nine narrower or wider lobules that were positioned opposite to centriolar microtubule blades (Fig. 3 B), like Cep57. When colabeled, nine signals of Cep57 and Cep63 were adjacent (Fig. S3 B), in agreement with Cep57 serving as a loading factor for Cep63 (Lukinavicius et al., 2013; Wei et al., 2020). On mature centrioles with more abundant Cep63 signal, individual Cep63 lobules were in contact, enclosing the space around the centriole. The linear pattern of Cep63 on longitudinally viewed centrioles was, hence, visible on some (Fig. S3 C), but not on all centrioles, even after expansion.

Finally, we analyzed the distribution of Plk4’s receptor Cep152. Cep152 associates with Cep63 through its C terminus (Brown et al., 2013; Kim et al., 2019) and with Plk4 through its outward extended N-terminus (Cizmecioglu et al., 2010; Dzhindzhev et al., 2010; Hatch et al., 2010; Park et al., 2014; Sonnen et al., 2013). We employed two antibodies targeting the middle region (Mid) and N-terminus (Nt) of Cep152 (Fig. S1 A). On mature centrioles, Cep152 signal formed an ∼500-nm-wide and ∼75-nm-thick cylinder (Fig. 3, A and E). The inner layer of the cylinder was ∼35–40 nm away from the centriole’s microtubules and its surfaces appeared rugged. On horizontally positioned centrioles, the signal appeared as an irregular meshwork and images of tilted centrioles showed that the mesh was three-dimensional (Fig. 3 E and Fig. S3 D). To further decipher the Cep152 pattern, we imaged maturing G1 centrioles, which are initially associated with less Cep152. Even at a lower density, the Cep152 pattern was irregular although the original and rotational averaging of three images showed signals, that could be roughly grouped into nine sections. Often, in each of the nine sections, two alternating areas of lower and higher densities were detected (Fig. 3 G, arrows). Immunolabeling with the Mid antibody occasionally revealed a ninefold pattern (Fig. 3 F). Lowering the cytoplasmic levels of Cep152 by siRNA, which deprived centrioles of Cep152 to various degrees (Fig. 3 H), failed to reveal its potential ninefold accumulation pattern. Even when most Cep152 was removed from centrioles, the remaining Cep152 Nt signal showed a similar pattern as before depletion, only of lower intensity. Our observation that Cep152 lacks a clear ninefold symmetry agrees with a previous report (Takao et al., 2019) and, thus, it likely reflects its natural molecular architecture. We also find it unlikely that the ninefold organization of Cep152 is even achievable. Its anchoring partner Cep63, which is localized closer to the centriole in the S phase, provides a wide platform for Cep152 loading (Fig. 3 B), and biochemical evidence suggests that Cep152 dimerizes and contains disordered domains (Kim et al., 2013). A more sophisticated analysis will be needed to decipher its molecular arrangement further. Nevertheless, we suggest that at the current level of detail, the molecular organization of Cep152 can explain the flexibility in the positioning of Plk4 and procentrioles around the mother centriole circumference in human cells (Fig. 2). It will be fascinating to explore whether such flexibility exists in other species, especially ones harboring narrower centrioles, such as *C. elegans* and *Drosophila*. Indeed, comprehensive molecular mapping of *C. elegans* centrosomal proteins (Woglar et al., 2022) shows that SPD2 (Cep192 homolog), a receptor for Zyg-1 (Plk4 homolog), organizes in an orderly, ninefold circular pattern around the mother centrioles. Similarly, in flies, Cep152 homolog Asterless (Asl; Varmark et al., 2007) organizes in a ninefold symmetry (Tian et al., 2021).

**Figure 4.**
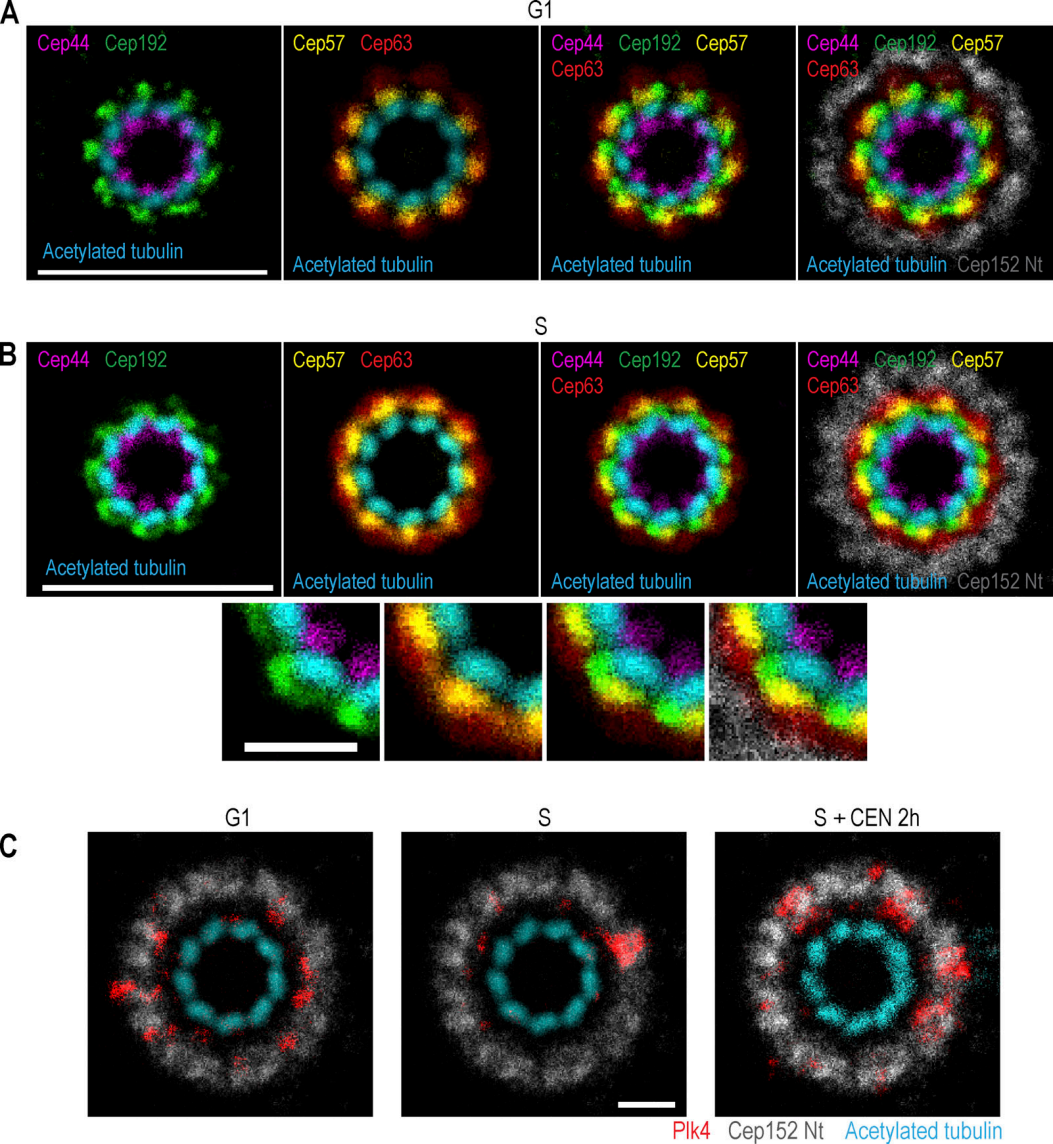
**The mutual position of Plk4 and a set of proximal centrosomal proteins in relation to mother centriole microtubules.** Logarithmically growing HeLa^C1-GFP^ cells were immunolabeled with antibodies to detect Plk4, Cep44, Cep192, Cep57, Cep63, or Cep152. Samples were expanded fourfold and immunolabeled for acetylated tubulin. Centrioles positioned vertically to the imaging plane were imaged using STED. Rotational images of individual proteins and acetylated tubulin were generated as described in Fig. S1 F and aligned in different combinations, as described in the text and in Materials and methods. Centriole microtubule blades were used as a reference during alignment. Proteins were assigned different colors for easier visualization. **(A)** Alignment of proximal centrosomal components from G1 centrosomes, characterized by lower amounts of Cep192, Cep63, and Cep152 in comparison to S phase cells. **(B)** Alignment of proximal centrosomal components from S phase centrosomes. **(C)** Alignment of centrosomal Plk4 signal with Cep152 and mother centriole microtubules. Plk4 signals are from G1 and S phase centrosomes and from S phase centrosomes treated with Plk4 inhibitor CEN. Scale bars: 2 µm in A and B, and 0.5 µm in B inserts and C.

To determine the position of each protein with respect to others, we aligned individual rotational images in different combinations. Images were flipped horizontally, if needed, to orient microtubules clockwise. Canvases of all images were adjusted so that the rotational centers of centrioles were in the middle of the canvas. Centrioles were then rotated around the centriole’s center to align centriole microtubule blades, the position of which was used as a reference for other proteins. After alignment, proteins were assigned a different color and their images were merged in different combinations. This generated a map depicting the mutual position of proteins on the centrioles of HeLa cells (Fig. 4).

**Figure 5.**
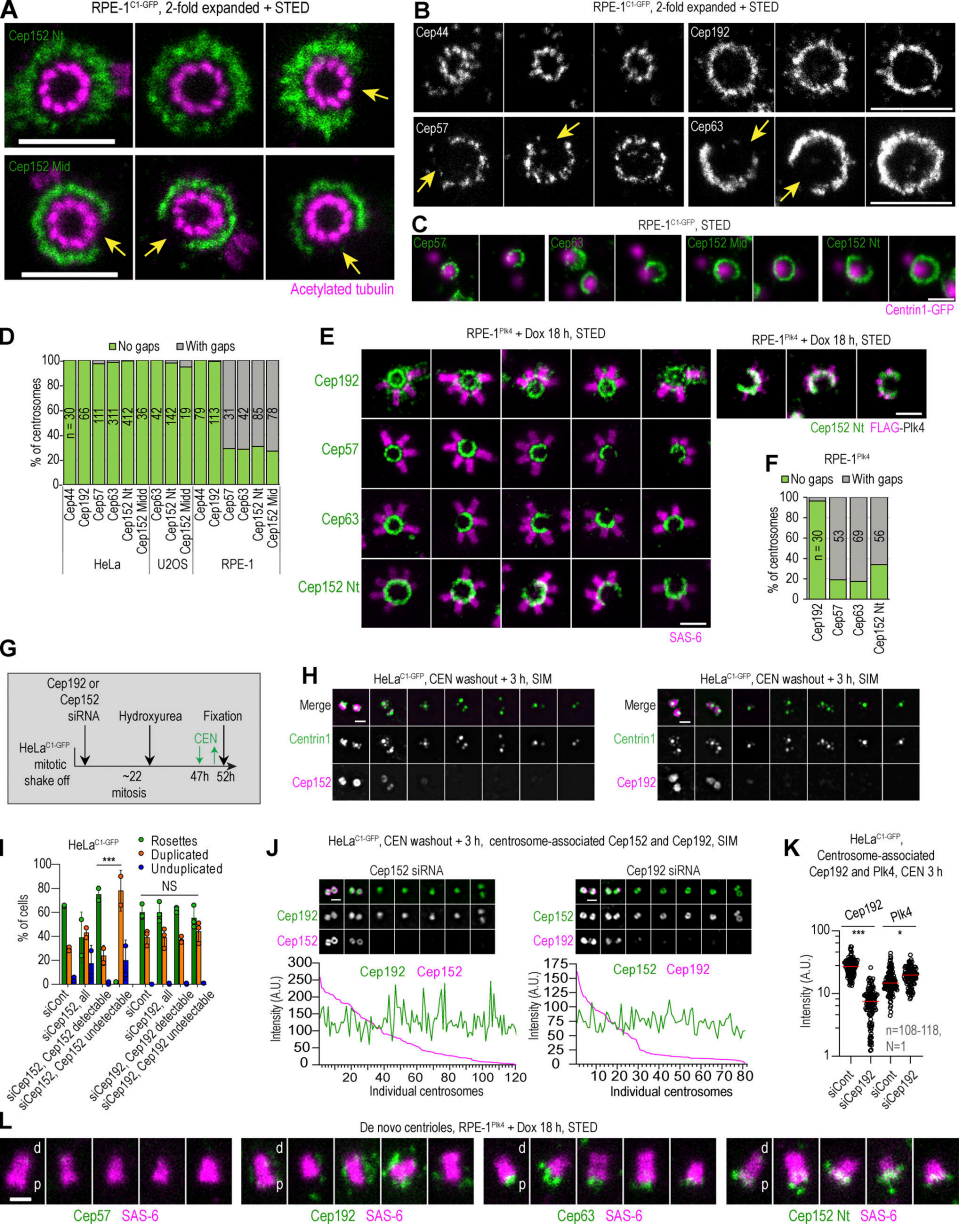
**Asymmetrical distribution of Cep152 in RPE-1 cells and its relationship with Cep192.** Logarithmically growing RPE-1^C1-GFP^ cells were immunolabeled for Cep44, Cep192, Cep57, Cep63, or Cep152. Cep152 was detected using an antibody against its N-terminus (Nt) or middle portion (Mid; Fig. S1 A). Some samples were additionally expanded twofold and labeled for acetylated tubulin. **(A)** The discontinuous appearance of Cep152 signals with gaps (arrows) extending over a variable number of centriole microtubule blades. Centrioles are shown in the top view. **(B)** The discontinuous appearance of Cep57 and Cep63 showing gaps (arrows). In contrast, Cep44 and Cep192 show continuous ring-like distribution. **(C)** Cep57, Cep63, and Cep152 signal in relation to Centrin1-GFP signal marking centrioles. Regardless of the gaps in Cep57, Cep63, and Cep152 signals, mother centrioles duplicate and regularly form only one daughter centriole. **(D)** Quantification of centrosomes with gaps in the signal of the indicated protein around the mother centriole. Centrioles imaged in vertical or near vertical orientation were quantified. **(E and F)** RPE-1^Plk4^ cells were treated with Dox for 18 h to induce overexpression of FLAG-Plk4 and formation of centriolar rosettes, and cells were immunolabeled for indicated proteins. **(E)** Centrosomes with centriolar rosettes to illustrate the absence of procentrioles at regions lacking Cep57, Cep63, and Cep152. Cep192 forms a complete ring around mother centrioles. Right: The absence of Plk4 signal on the regions of the centrosome lacking Cep152. **(F)** Quantification of centrosomes in RPE-1^Plk4^ cells exhibiting gaps in the signals of Cep192, Cep57, Cep63, and Cep152. Centrioles imaged in vertical or near vertical orientation were quantified. **(G)** Experimental strategy used in H–K (for more details, please see Materials and methods). **(H)** The effect of Cep192 or Cep152 depletion on the assembly of centriolar rosettes. Cells were depleted for Cep192 or Cep152, and centriolar rosettes were induced by transient treatment with CEN and Cep192 and Cep152 were immunolabeled. Centrin1-GFP marks centrioles. **(I)** Quantification of cells from H containing centriolar rosettes, duplicated centrioles, or at least one unduplicated centriole. 200 cells were counted per condition in each experiment. **(J)** The effect of Cep192 or Cep152 depletion on Cep152 and Cep192 centrosomal levels. Intensities of Cep192 and Cep152 were determined from widefield recordings and plotted for individual centrosomes. **(K)** The effect of Cep192 depletion on centrosomal Plk4 levels. Plk4 and Cep192 were labeled and their centrosomal intensity was quantified from widefiled images. Red lines: average intensity. **(L)** Localization of Cep57, Cep192, Cep63, Cep152, and SAS-6 on de novo–formed centrioles in S phase RPE-1^Plk4^ cells treated with Dox for 18 h. The wider end of the SAS-6 signal distinguishes the centriole’s proximal (p) end from the distal (d) end. Cep192 associates with the centrioles laterally, Cep63 and Cep152 associate with the centriole’s proximal ends, while Cep57 does not associate with de novo formed centrioles. Scale bars: 0.5 µm for STED, 1 µm for twofold expansion/STED, and 2 µm for widefield.

We next questioned if our map represents centrosomes from other human cell lines. So, we tested two additional human cell lines, U2OS and RPE-1, which are routinely used for centrosome studies. We were specifically interested in whether the Cep152 pattern observed in HeLa cells is preserved across cell lines. In U2OS cells, Cep63 and Cep152 showed a similar pattern as in HeLa cells (Fig. S3 E). Surprisingly, in RPE-1 cells, we detected numerous centrosomes with an interrupted ring of Cep152, regardless of whether we used Cep152 Nt or Mid antibody (Fig. 5 A). The gaps in the Cep152 signal were of variable size, ranging from small gaps to gaps spanning half of the centriole’s circumference. Where Cep152 was localized, it was organized in a similar pattern as in HeLa and U2OS cells. Further analysis of RPE-1 centrosomes showed that Cep57 and Cep63 exhibited similar gaps, while Cep192 and Cep44 reproducibly localized in closed rings (Fig. 5, B and C). Quantification of mature centrioles showed that in HeLa and U2OS cells, gaps in Cep57, Cep63, and Cep152 were rarely present, while they were prevalent in the population of RPE-1 centrosomes (Fig. 5 D). Young immature centrioles in G1, which are still recruiting these proteins, were excluded from quantification. Notably, despite the gaps in Cep152, mechanisms instilling the centriole block to reduplication are still intact in RPE-1 cells as we did not observe any errors in centriole copy number. We additionally explored the positioning of procentrioles within centriolar rosettes in Plk4-overexpressing RPE-1^Plk4^ cells (Fig. 5 E), which also exhibit gaps in Cep57, Cep63, and Cep152 (Fig. 5, E and F). Procentrioles exclusively formed around the parts of the centrioles associated with Cep57, Cep63, and Cep152. Consistently, the regions lacking Cep152 lacked Plk4 signal (Fig. 5 E, right panel).

Although it is unclear to us why some areas around centrioles in RPE-1 cells lack Cep152 scaffolding factors, this naturally occurring phenomenon is informative because it shows that Cep192 may not be critical for Cep152 and Plk4 recruitment since neither protein was detected at regions containing Cep192 but lacking Cep57 and Cep63. To explore this possibility, we depleted Cep152 or Cep192 following the scheme in Fig. 5 G. siRNA was transfected in G1, and cells were allowed to complete one mitosis before they were trapped in their second S phase by hydroxyurea (HU). After 47–48 h of transfection (∼6 h into the S phase arrest), cells were either fixed or treated with CEN for 2 h to induce Plk4 accumulation and procentriole rosettes after their washout. To ensure that the mother centriole structure was not compromised by Cep152 or Cep192 depletion, we allowed only one mitotic division to occur after transfection of siRNA and analyzed the formation of centriolar rosettes within ∼50 h of siRNA treatment. In many cells, we could deplete Cep192 and Cep152 and use partially depleted cells as internal controls. Depletion of Cep152, but not Cep192, inhibited procentriole rosette formation and increased the number of cells with unduplicated centrioles (Fig. 5, H and I). In addition, colabeling of Cep192 and Cep152 after their separate depletion showed that their centrosomal localization is largely independent (Fig. 5 J), at least at the level of depletion achieved in our experiments. Cep192-depleted centrioles were efficient in Plk4 accumulation after CEN addition (Fig. 5 K).

Finally, localization of Cep192 on procentrioles that formed de novo in the cytoplasm of Plk4-overexpressing RPE-1^Plk4^ cells (Fig. 5 L) additionally suggests that Cep192 may not be directly required for procentriole initiation. While Cep63 and Cep152 localize around and below the proximal end of the cartwheel (identified as the wider end of the SAS-6 signal), Cep192 localizes to de novo centrioles laterally along elongated SAS-6 signals, which is more consistent with its role in procentriole structuring than initiation.

Our analysis provides novel perspectives on the molecular localization of Plk4 and its behavior, as well as the centrosomal organization of proximal proteins that mediate Plk4 localization. We show that in both G1 and S phases, Plk4 occupies a cylindrical space around the proximal end of mother centrioles and localizes as discrete, irregular signals variably distanced from each other (Fig. 1, A and B), rather than forming a continuous Plk4 ring. In early S phase, procentriole-bound Plk4 is organized in an ∼100 nm wide focus which, as previously shown (Kim et al., 2013), is usually of higher intensity than surrounding Plk4 signals. Total centrosomal levels of Plk4, on average, are lower in S than in the G1 phase (Fig. 1 D). Thus, the procentriole-unbound fraction of Plk4 must be reduced during the G1 to S transition. We also found that in S phase, the levels of procentriole-unbound Plk4 vary several-fold amongst centrosomes within the same population, suggesting that duplicated centrosomes can tolerate variable levels of procentriole-unbound Plk4 while still maintaining the block to centriole reduplication. Since it has been reported that a modest, twofold elevation in the levels of centrosomal Plk4 can result in substantial centrosome amplification in some mouse tissues (Levine et al., 2017), we speculate that factors other than Plk4 stoichiometry, such as its posttranslational modifications and association with other molecules likely influence centriole copy number, either intrinsically within centrosomes or by some other cell type-specific manner. Another intriguing finding from this work is that the centrosomal Plk4 receptor Cep152 organizes a complex platform around mother centrioles, which lacks a clear ninefold organization, that is permissive for Plk4 loading and procentriole initiation. We provide evidence that both Plk4 foci and procentrioles can form at variable positions in relation to the mother centriole microtubules and under variable distances from each other around the mother centriole circumference. While the requirement for such flexibility may not be evident in cycling cells that generate only one procentriole per cell cycle, it could be utilized during multiciliation, particularly in cells that produce fewer cilia and generate multiple centrioles in association with mother centrioles, such as in olfactory epithelium (Ching and Stearns, 2020). Whether centrosomes of other organisms, especially the narrower ones built of microtubule singlets and doublets, have similar flexibility would require further ultrastructural studies.

Two mathematical models have been put forward to explain the change in Plk4 distribution at the G1/S phase transition and the regulation of centriole copy number. Leda et al. (2018) postulate that Plk4 equally accumulates in nine compartments around mother centrioles in G1, which can evenly exchange their content via diffusion. After the formation of Plk4/STIL complexes in S phase, Plk4/STIL first symmetrically accumulates in all compartments, each of which exchanges Plk4/STIL and competes for their cytoplasmic pool. Then, stochastically, one compartment eventually solidifies as the winning compartment with high Plk4 activity. The levels of Plk4 in other compartments are then kept low due to Plk4 degradation, lack of Plk4 retention, and competition for cytoplasmic Plk4/STIL complexes. It would be interesting to see how this model behaves using the unequal and discontinuous Plk4 distribution we have documented in G1 cells.

Another model by Takao et al. (2019) is based on the inherent property of Plk4 to self-organize into macromolecular condensates in vitro (Montenegro Gouveia et al., 2018; Park et al., 2019; Yamamoto and Kitagawa, 2019). The model also postulates that Plk4 localizes to 12 slots, driven by the 12-fold symmetry of Cep152, which then organizes into six compartments. Importantly, the model dictates that one of the six compartments already contains most Plk4 before the centrosomal binding of STIL. In S phase, binding of STIL would occur at all compartments, further activating Plk4. However, the dominant Plk4 site generates more active Plk4, which dissociates to phosphorylate Plk4 in other compartments, leading to its degradation or dissociation from the centrosome. Our imaging data argue against a systematic presentation of one dominant Plk4 focus on G1 centrosomes, so we are not in favor of the idea that the symmetry breaking of Plk4 occurs before the centrosomal loading of STIL. We could not confirm the 12-fold symmetry of Cep152 and the 6-fold symmetry of Plk4, consistent with the fact that after Plk4 overexpression or its transient catalytic inhibition, greater than six centrioles can form around mother centrioles.

Interestingly, notwithstanding the differences in the initial number of Plk4 compartments (which, based on our data may not be relevant) and other computational differences, both models exploit distinctive behavior of differently phosphorylated Plk4 and STIL in terms of their turnover (Arquint et al., 2018; Cunha-Ferreira et al., 2009; Guderian et al., 2010; Holland et al., 2010), Plk4 activity (Arquint et al., 2015; Moyer et al., 2015), and retention rate on the centrosome (Arquint et al., 2015; Arquint and Nigg, 2014; Kim et al., 2016; Ohta et al., 2014; Yamamoto and Kitagawa, 2019). We hope that our ultrastructural analysis of centrioles and Plk4 behavior under various conditions will help further advance existing models and provoke new ones to advance our understanding of the phenomenon of centriole duplication.

## Materials and methods

### Cell lines and their maintenance

Human HeLa^C1-GFP^ (Piel et al., 2000) and RPE-1^C1-GFP^ (Uetake et al., 2007) cell lines constitutively express Centrin1-GFP (C1-GFP). RPE-1^Plk4^ cells harbor Dox-inducible mouse Plk4 gene (Evans et al., 2021) fused with FLAG epitope tag on its N-terminus (kindly provided by Dr. Andrew Holland at Johns Hopkins University, Baltimore, MD, USA). The parental cell line used to generate RPE-1^Plk4^ cells constitutively expresses FLAG-tagged Cas9 (Lambrus et al., 2016). Human U2OS cells were from ATCC. All cells were grown in DMEM media supplemented with 1% penicillin/streptomycin and 10% of fetal bovine serum (FBS). For RPE-1^Plk4^ cells, tetracycline-free FBS was used. Cells were incubated in a humidified environment at 37°C and 5% CO_2._ For various microscopy analyses, cells were plated on round 25 or 18 mm 1.5 (0.17 mm)-thick high tolerance cover glasses (64-0735 and 64-0714; Warner Instruments).

### Cell synchronization and cell treatments

Cells were synchronized by mitotic shake-off from tissue culture flasks containing logarithmically growing cells by gently tapping the flasks after removing ∼90% of the growing media. Mitotic cells were then replated onto fresh Petri dishes or glass coverslips, as needed. After replating, cells typically exit mitosis within 1 h, reach the S phase after ∼9 h, the G2 phase after ∼16–18 h, and enter mitosis after ∼18–22 h (Shukla et al., 2015). To arrest cells in early S phase, 3 mM HU (H8627; Sigma-Aldrich) was added to G1 cells (usually 2 h after mitotic shake-off) or to logarithmically growing cell cultures. Plk4 was inhibited by adding 0.1 μM CEN (5687; Tocris) to the culture media for the time indicated in the figure panels. An equal volume of the drug solvent DMSO (D2650; Sigma-Aldrich) was used as a control.

To analyze centrosomal Plk4 in control and CEN-treated G1 and S phase cells, HeLa^C1-GFP^ cells were replated on glass coverslips after mitotic shake-off and treated with CEN or DMSO for 2 h while in the G1 (5 h after shake-off) or early S phase (11 h after shake-off) and fixed 2 h later. To examine the effect of CEN treatment on centrosomal Plk4 in S phase-arrested cells, logarithmically growing HeLa^C1-GFP^ were treated with HU for 16 h and treated with CEN for up to 10 h before fixation. To generate centriolar rosettes for microscopy observation, cells were treated with CEN for 2–3 h, CEN was washed, and cells were fixed 3 h later. To induce expression of FLAG-Plk4, RPE-1^Plk4^ cells were treated with 0.5 µg/ml Dox for the time indicated in the figure panels.

### Immunoblotting

Total cell extracts in Laemmli buffer supplemented with protease and phosphatase inhibitors were denatured and loaded onto polyacrylamide gels (6–7% as needed) for separation. Proteins were transferred to PVDF membranes and blocked in 6% milk in TBST buffer (Tris-buffered saline pH 7.5 with 0.1% tween-20). Primary antibodies were diluted in TBST with 3% milk and incubated at 4°C overnight. Membranes were washed in TBST and incubated with HRP-conjugated secondary antibody in TBST with 3% milk for 1 h at RT. After washing, signals were detected using Clarity western ECL substrate (170-5060; BioRad). For the detection of endogenous Plk4, SuperSignal West Femto (34094; Thermo Fisher Scientific) was added to the Clarity ECL substrate to 10% of the total volume. Antibodies were used as follows: SAS-6 (sc-81431; Santa Cruz) at 1:2,000, mouse anti-FLAG (F1904; MilliporeSigma) at 1:2,000, and mouse anti-Plk4 (MABC544; MilliporeSigma) at 1:500. HRP-conjugated secondary anti-mouse and anti-rabbit antibodies (NA931S and NA934VS, respectively; Amersham) were used at 1:10,000.

### Quantification of immunoblot signals

Intensities of individual bands were measured from inverted, 8-bit images using Fiji (National Institute of Health; NIH). Background levels of the same surface area were subtracted from the signals. Band intensities were normalized to ponceau-stained membranes.

### siRNA depletion

The specificity of Plk4 antibodies was evaluated after siRNA depletion of Plk4. HeLa^C1-GFP^ cells were transfected with non-targeting siRNA (5′-UGG​UUU​ACA​UGU​CGA​CUA​A-3′; Dharmacon) or Plk4 siRNA (5′-GAA​AUG​AAC​AGG​UAU​CUA​A-3′; Dharmacon) using Oligofectamine (12252011; Invitrogen) following the vendor’s instructions. Cells were collected for immunolabeling after 40 h of siRNA depletion.

To deplete Cep152 and Cep192, G1 HeLa^C1-GFP^ cells were treated with Cep192 siRNA (5′-GCU​AGU​AUG​UCU​GAU​ACU​UGG-3′; Dharmacon), Cep152 siRNA (sc-90225; Santa Cruz), non-targeting siRNA (5′-UGG​UUU​ACA​UGU​CGA​CUA​A-3′; Dharmacon), or control siRNA-A (sc-37007; Santa Cruz) 2 h after shake-off as described above. Cells were treated with HU 27 h after shake-off, while in their second G1, and fixed in their second S phase 48 h after treatment with siRNA. In some experiments, ∼48 h after shake-off, cells were treated with CEN for 2 h to induce Plk4 accumulation and fixed, or they were treated with CEN transiently to allow the formation of centriolar rosettes after CEN washout (as depicted in Fig. 5 G).

### Immunofluorescence

Cells were fixed in 1.5% formaldehyde in PBS at RT for 4 min and post-fixed in 100% methanol at −20°C for 4 min followed by rehydration in PBS. Prior to immunolabeling, all samples were blocked in immunofluorescence (IF) buffer (1% BSA [A9647; Sigma-Aldrich] and 0.05% Tween-20 [P9416; Sigma-Aldrich] in PBS) for 15 min at RT. Cells were then incubated with primary antibody diluted in IF buffer at 37°C for 2 h or at 4°C overnight. After washing with PBS, cells were exposed to secondary antibody diluted in IF buffer and incubated at 37°C for 1.5 h. DNA was visualized by incubating with Hoechst (H3570; Thermo Fisher Scientific) diluted 1:1,000 in PBS.

The following primary antibodies were used: rabbit anti-Plk4AH01 at 1:500, rabbit anti-Plk4AH03 at 1:500 (Levine et al., 2017; Moyer et al., 2015; all kindly provided by Dr. Andrew Holland), rabbit anti-Plk4Oeg (Wong et al., 2015) at a final concentration of 1 µg/ml (kindly provided by Drs. Karen Oegema and Arshad Desai at the University of San Francisco, San Francisco, CA, USA), mouse anti-SAS-6 (sc-81431; Santa Cruz) at 1:200, rabbit anti-CP110 (2780-1-AP; Protein-tech) at 1:1,000, rabbit anti-Cep152-Nt (A302-479A; Bethyl) at 1:3,000, rabbit anti-Cep152-Mid (A302-480A; Bethyl) at 1:1,000, rabbit anti-Cep192 (A302-324A-M; Bethyl) at 1:500, rabbit Cep57 (GTX115931; GeneTex) at 1:300, rabbit anti-Cep44 (24457-1-AP; Protein-tech) at 1:800 (or 1:700 for expansion), rabbit anti-Cep63 (06-1292; Millipore Sigma) at 1:1,000 (or 1:700 for expansion), and mouse anti-Cep63 (TA809276S; OriGene) at 1:300. For the Cep192 siRNA and Cep152 siRNA experiments, Cep192 and Cep152 antibodies were directly labeled with CF555 (92274; Biotium labeling kit), Alexa Fluor 594 (A20004; Thermo Fisher Scientific), and Aberrior STAR RED dyes (43354; Millipore Sigma), and used at 1:100.

Secondary antibodies: Anti-mouse or anti-rabbit conjugated with Aberrior STAR RED or STAR ORANGE dyes were used at 1:200–600 dilution (STRED-1002 STORANGE-1002, STRED-1001, STORANGE-1001; Abberior). Anti-rabbit conjugated with CF568 (20801; Biotium) was used at 1:800 dilution. Single-domain anti-rabbit antibodies FluoTag-X4-N2404-AbRED (N2404-AbRED-S; NanoTag Biotechnologies) were used at 1:500 dilution.

### Sample expansion

Cells growing on round glass coverslips were immunolabeled for Plk4 or other indicated centrosomal proteins and detected using secondary antibodies conjugated with STAR RED dye, which is preserved during sample expansion, allowing direct detection of preimmunolabeled proteins after expansion (Vásquez-Limeta et al., 2022). Samples were postfixed in 4% formaldehyde in PBS at RT for 1 h and incubated at 40°C for 16 h in a solution containing 30% acrylamide (A4058; Sigma-Aldrich) and 4% formaldehyde in 1 × PBS. Following three 10-min PBS washes, coverslips were placed on a parafilm-covered Petri dish floating in an ice-water bath. Precooled gelling mixture (20% acrylamide, 7% sodium acrylate [S03880; Pfaltz & Bauer], 0.04% bis-acrylamide [A9926; Sigma-Aldrich], 0.5% ammonium persulfate [248614; Sigma-Aldrich], and 0.5% tetramethylethylenediamine [411019; Sigma-Aldrich]) was pipetted to the coverslips and incubated on ice for 20 min and then 1 h at RT. After polymerization of the gel, several punches were excised using a 4-mm biopsy puncher (33–34-P/25; Integra Miltex). Punches were placed in an empty 50-ml conical tube and dry-heated at >90°C for 10 min. Preheated SDS solution (200 mM SDS, 200 mM NaCl, and 50 mM Tris, pH 9.0) was then added to the punches, and the punches were boiled for 1 h at >90°C. The content of the tube was cooled to RT and SDS was removed by exchanging PBS every 20 min for the first 2 h, followed by an overnight wash in PBS at 4°C. To immunolabel acetylated tubulin, punches were blocked in IF buffer (1% BSA and 0.05% Tween-20 in PBS) for 1 h at RT and incubated with anti-acetylated tubulin antibody (T7451; Sigma-Aldrich) at 1:3,000 in IF buffer for 48 h at 4°C. Punches were washed in PBS for 1 h and incubated with anti-mouse antibody conjugated with Abberior STAR ORANGE (STORANGE-1001; Abberior) diluted to 1:50 for 24 h at 4°C in IF buffer. DAPI (5 mg/ml) was added to 1:1,000 during immunolabeling with a secondary antibody to visualize DNA. Punches were washed in PBS and either imaged in PBS (providing an expansion factor of twofold) or expanded in deionized H_2_O (providing an expansion factor of fourfold), and mounted in Rose chambers for imaging, as detailed in Kong and Loncarek (2021).

### Microscopy

#### Widefield fluorescence microscopy

Images were acquired using a Nikon Eclipse Ti inverted microscope, equipped with an ORCA-Flash4.0 V3 (Hamamatsu) and Intensilight C-HGFIE illuminator, using 100× NA 1.42 Plan Apo objective with 1.5× magnifying tube lens. 200-nm thick Z-sections spanning the entire cell or entire centrosome, as needed, were acquired.

#### Structured illumination microscopy (SIM)

SIM was performed on N-SIM, Nikon Inc., equipped with 405, 488, 561, and 640 nm excitation lasers, Apo TIRF 100× NA 1.49 Plan Apo oil objective, and back-illuminated 16 µm pixel EMCCD camera (DU897; Andor). 100-nm-thick Z sections were acquired in 3D SIM mode and reconstructed.

#### 2D stimulated emission depletion (STED) microscopy

Imaging was performed with STEDYCON (Abberior Instruments) assembled on Eclipse Ti2 inverted microscope (Nikon Inc.) using 100×, NA 1.45 Plan Apo objective. Avalanche photodetectors (650–700 nm; 575–625 nm; 505–545 nm; DAPI detection) were used to detect the signals. Browser-based control software (Abberior Instruments) was used to generate STED images. Images were acquired with a pinhole size of 32–64 μm and a pixel size of 10 nm. Excitation lasers for STAR RED and STAR ORANGE were run with 2–10% laser power and depleted with the STED laser at 97.88% and 100%, respectively. Signals were detected within a 7-ns gate. In some images, the position of centriole-associated Centrin-GFP was detected using predetermined software parameters for Alexa Fluor 488.

#### Electron microscopy

Cells growing on glass coverslips were fixed in 2.5% glutaraldehyde and 0.25% formaldehyde in PBS (pH 7.4) for 1 h at RT, washed in PBS for 30 min (10 min each wash), prestained with 1% osmium tetroxide, and 1% uranyl acetate, dehydrated in graded ethanol series, and then embedded in EMbed-812 resin. 80-nm-thick serial sections were sectioned, transferred to the formvar-coated copper grids, and stained with uranyl acetate and lead citrate. Samples were imaged using an FEI Tecnai 12 Spirit transmission electron microscope operating at 80 kV. Image analysis was performed in Adobe Photoshop and Fiji (NIH).

### Image acquisition and analysis

Within one experiment, images were acquired under identical imaging settings. The levels of fluorescent signals were sometimes differentially adjusted on final image panels to improve the visibility of dimmer signals. Such adjustments were not performed individually if a quantitative comparison between centrosomes was intended. Maximum intensity projections of all acquired Z slices spanning a part of the cell containing centrioles are presented for widefield imaging. Fiji (NIH) and NIS-Elements (Nikon Inc.) were used for image analysis and assembly.

### Rotational images and superposition of rotational images

To generate rotational images, recordings of centrioles oriented vertically to the imaging plane were used. Images of centrioles were centered so that the physical center of a centriole was placed in the middle of the image canvas. Two rotational images of 0°, 40°, and 80° were generated and assembled into a stack, and the average intensity projection was made and used as the rotational image (Fig. S1 F). Images were rotated at a 40° increment to reveal a potential ninefold pattern of Plk4 and other proteins (40° × 9 = 360°). Averaging more than three rotational images was strictly avoided to not generate artificial ninefold symmetry by excessive rotation of the brightest Plk4 foci.

To illustrate the mutual arrangement of various centrosomal proteins, each protein was coimmunolabeled with acetylated tubulin and two-color STED images of the centrioles oriented vertically to the imaging plane were recorded. Rotational images were then generated as described above. Using the Transform tool in Fiji, images of centrioles were flipped horizontally if needed so that their centriolar microtubules showed a clockwise turn (as it appears when centrioles are viewed from the distal end). Further, images of centrosomes were rotated around the centriole’s center until one of the centriole’s microtubule triplets was facing a 12 o’clock position. Each centrosomal protein of aligned centrosomes was then differently color coded, and their images were assembled in stacks in different combinations and projected.

### Definition of a Plk4 focus

For the quantification of Plk4 foci numbers, we considered a single Plk4 signal as a focus if its integrated density was greater than equal to that of the minimal density of procentriole-associated Plk4 in a population of control S-phase centrosomes measured in a circle of 180 nm diameter.

### Fluorescence intensity quantification of centrosome-associated proteins

Quantifications were performed on widefield or STED images. For widefield images, Z stacks of 200-nm-thick sections spanning the entire centrosome were acquired and maximum intensity projections of all Z slices were generated. For each centrosome-associated protein, the integrated density of signals was measured within a defined area of a constant size encircling the centrosome or centrosomal region of interest. The background intensity of an equivalent area proximal to each centrosome was measured and subtracted from the signal intensity. Area size was dependent on the signal being measured. Integrated density measurement of Plk4 signals from STED images (for Fig. 1 D and Fig. S2 E) were measured within a circle of a diameter of 60 pixels for total centrosomal signal or 20 pixels for procentriole-associated Plk4 or Plk4 foci in CEN-treated cells (pixel size = 10 nm). The intensity and the size of Plk4 foci were analyzed using a plot profile in Fiji after drawing a line through the center of the focus. Intensity measurements along the length of the line were recorded and averaged for multiple foci. The average diameter of Plk4 signals in Fig. 1 K was determined by measuring the length of the averaged signal at half-width–half-maxima.

### Angles between procentrioles and Plk4 foci and their relation to parental centriole microtubules

To classify the position of procentrioles and Plk4 foci as opposite to the microtubule blade (O) or opposite the gap between adjacent microtubule blades (B), we used images of centrosomes with parental centrioles in orthogonal or near-orthogonal orientation to the coverslip. Centrioles were first selected in widefield mode. Their orientation was reviewed by focusing on the centrosome, and only centrosomes that exhibited no visible tilt were imaged. After 2D STED imaging, only centrioles without distortion of acetylated tubulin were used for measurements. Two lines were drawn from the physical center of the parental centriole toward the proximal outer ends of procentriole microtubules or outside the Plk4 focus (Fig. 2), and the determination was made whether a procentriole or Plk4 focus belonged closer to O or B category.

For the angle between two adjacent procentrioles or Plk4 foci, centrioles were imaged in orthogonal or near-orthogonal orientation, as described in the above paragraph. In some experiments, three Z sections were recorded by STED (Fig. 2, D and E), and in some experiments, Plk4 was colabeled with Cep152 and CP110 to confirm the orthogonal or near-orthogonal orientation of mother centrioles together with C1-GFP signals. To measure angles between two signals/procentrioles, two lines were drawn from the physical center of the parental and the centers of adjacent Plk4 foci/procentrioles, and the angle between the two lines was measured (Fig. 2). Cep44 and centriole acetylated tubulin signals (which organize in ninefold symmetry) were used as a control to test our strategy. Angles between their adjacent signals reproducibly grouped around 40° (Fig. 2 H).

### Statistical analysis

Statistical difference between two sets of data was determined in Excel using an unpaired, two-tailed Student’s *t* test with equal or non-equal variance, as determined using an F-test. NS, nonsignificant; *P ≤ 0.05; **P ≤ 0.01; and ***P ≤ 0.001. Dot plots and line plots were generated in Prism. Dot plots show all data points and red lines show average values. Sample sizes (the number of counted or measured centrioles/cells [*n*] and number of experimental replicates [*N*]) are indicated on the figure panels. The number of performed experiments can be found in the supplemental text at the end of the PDF.

### Online supplemental material

Fig. S1 shows epitopes of used antibodies, specificity of Plk4 antibodies, centrosomal Plk4 distribution, and a method used for generating rotational images. Fig. S2 shows Plk4 and procentriole organization in RPE-1 cells overexpressing Plk4 with or without CEN and centriolar rosettes in RPE-1 and HeLa cells. Fig. S3 shows images of Cep44, Cep63, Cep57, and Cep152 in HeLa and U2OS cells. The number of performed experiments can be found in the supplemental text at the end of the PDF.

## Supplementary Material

SourceData F1 is the source file for Fig. 1.

## Data Availability

Data are available from the corresponding author upon reasonable request.
