# Supplementary material for: Centrosomal organization of Cep152 provides flexibility in Plk4 and procentriole positioning
Source: J Cell Biol. 2023 Sep 14;222(12):e202301092. doi: 10.1083/jcb.202301092 (PMC10501443; doi:10.1083/jcb.202301092)
Supplement: SourceData F1 — is the source file for Fig. 1. [file JCB_202301092_SourceDataF1.pdf]

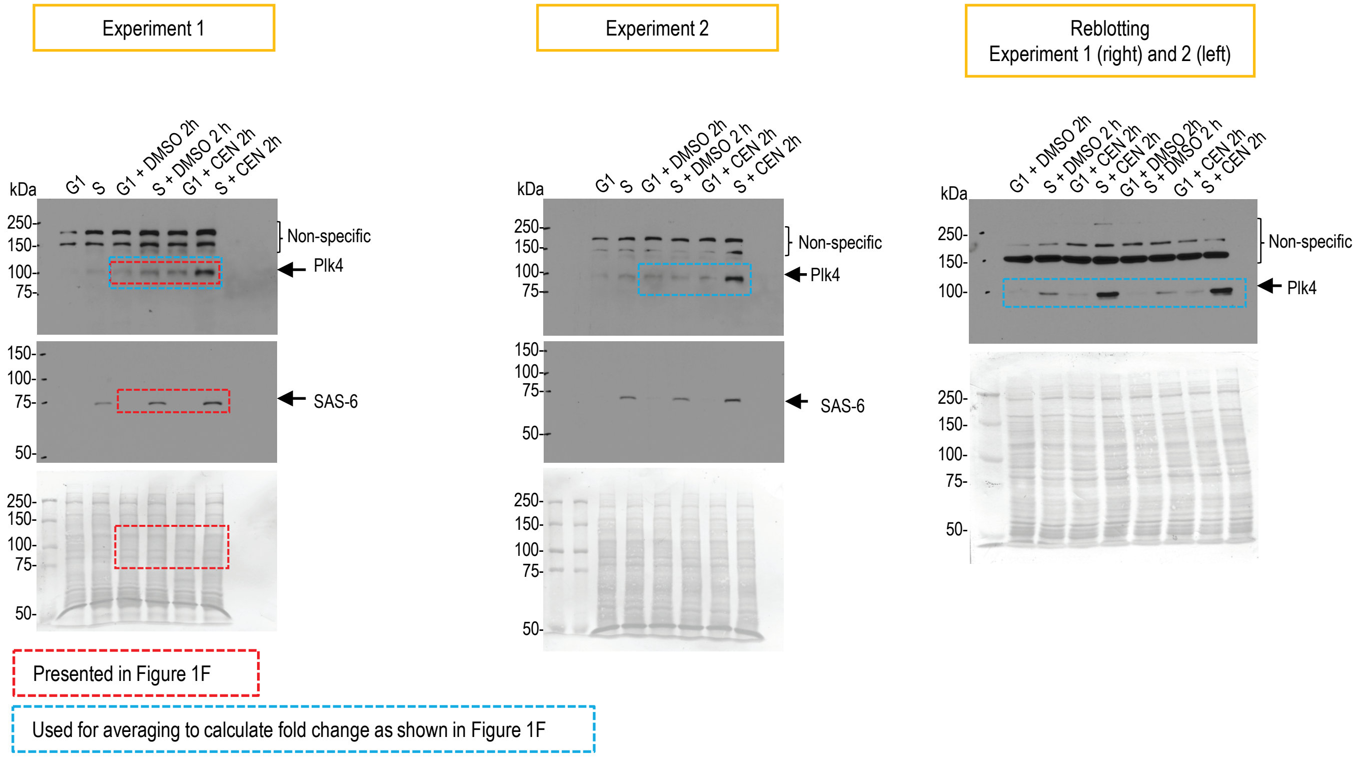

Source data for Plk4 in Figure 1E. Uncropped immunoblots and corresponding membranes stained with Ponceau after immunoblotting. G1 or S HeLa$^{C1\text{-}GFP}$ cells were treated with DMSO or centrinone (CEN) for 2 h and total cell lysate was probed with mouse anti-Plk4 and subsequently mouse anti-SAS-6 antibodies. Red boxes indicate the cropped regions presented in Figure 1E. Blue boxes indicate bands used for averaging and calculation of fold change shown in Figure 1E.

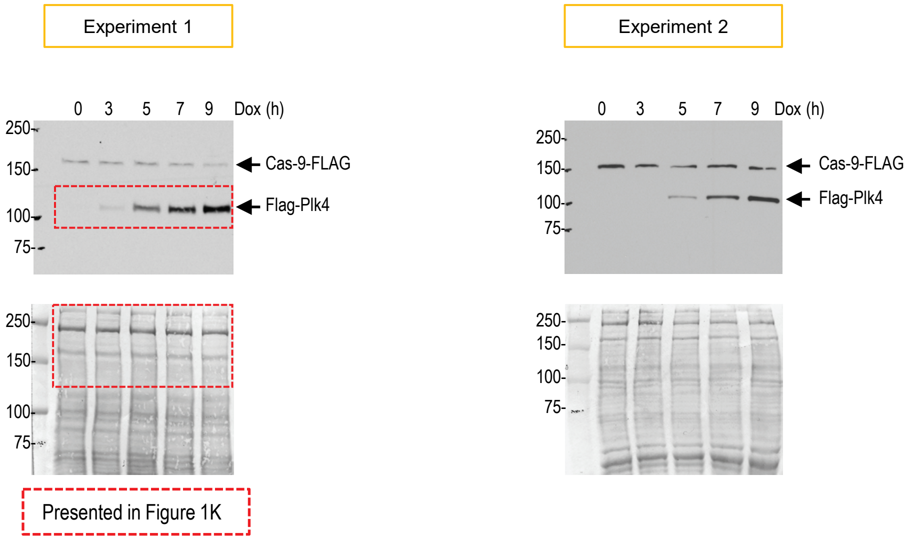

Source data for Figure 1J. Uncropped immunoblots and corresponding membranes stained with Ponceau after immunoblotting. S phase arrested RPE-1^{Plk4} were treated with doxycycline (Dox) to induce expression of FLAG-Plk4. Levels of FLAG-Plk4 after 3, 5, 7, and 9 h of doxycycline exposure were detected from total cell lysates using an anti-FLAG antibody. Red boxes indicate the cropped regions of presented in Figure 1J
